# Perspectives of Latinx Individuals Who Were Unvaccinated And Hospitalized for COVID-19: A Qualitative Study

**DOI:** 10.1001/jamanetworkopen.2022.18362

**Published:** 2022-06-17

**Authors:** Lilia Cervantes, Cynthia A. Hazel, Diana Mancini, Rocio I. Pereira, Laura J. Podewils, Sarah A. Stella, Joshua Durfee, Alana Barshney, John F. Steiner

**Affiliations:** 1 Department of Medicine, Denver Health and Hospital Authority, Denver, Colorado; 2 Department of Medicine, University of Colorado, Aurora; 3 Omni Institute, Denver, Colorado; 4 Office of Research, Denver Health and Hospital Authority, Denver, Colorado; 5 Institute for Health Research, Kaiser Permanente Colorado

## Abstract

**Importance:**

Latinx individuals in the United States have lower COVID-19 vaccination rates and higher rates of COVID-19 infections, hospitalizations, and deaths than non-Latinx White individuals. Little is known about the perspectives of Latinx adults who had not received the COVID-19 vaccination and were hospitalized for COVID-19.

**Objective:**

To describe the perspectives of Latinx individuals who were unvaccinated and subsequently hospitalized for COVID-19.

**Design, Setting, and Participants:**

This qualitative study was conducted using semistructured phone interviews with 25 Latinx adults who were unvaccinated and survived a COVID-19 hospitalization in a public safety net hospital in Colorado from February to November 2021. Interviews were audio recorded, and transcripts were analyzed using thematic analysis.

**Main Outcomes and Measures:**

Themes and subthemes of perspectives on vaccination.

**Results:**

Among 25 adults (14 [56.0%] women, 11 [44.0%] men; mean [SD] age, 51 [15] years) who participated, all participants self-identified as Latino, Latina, or Latinx or Hispanic. There were 11 individuals who relied on emergency Medicaid (hospital coverage for Denver residents who are undocumented), while 10 individuals (40.0%) were essential workers and 13 individuals (52.0%) were unemployed. In interviews, 3 themes (with subthemes) were identified: factors associated with vaccination after hospitalization (subthemes: fear of death, avoiding hospitalization and reinfection, convinced COVID-19 is real, and responded to pressure from others), concerns about the COVID-19 vaccine (subthemes: experimental status and short timeline for production, contents of vaccine unknown or concerning, vaccine considered ineffective, worrisome immediate and long-term adverse effects, mixed and conflicting information, and government aimed to control or mark population through vaccination), and opportunities to improve vaccine uptake (subthemes; sharing personal experiences through social media, testimonials about minimal vaccine adverse effects, connecting with friends and family about the hospitalization experience, making the vaccine more accessible, and connecting with trusted sources of information).

**Conclusions and Relevance:**

This study found that Latinx individuals who were unvaccinated and subsequently hospitalized for COVID-19 were motivated to engage in advocacy to encourage vaccination in their communities. These findings suggest that supporting patient advocacy after hospital discharge and continued efforts to create low-barrier, patient-informed public health strategies may be associated with increased vaccine uptake in Latinx communities.

## Key Points

**Question:**

What are the perspectives of Latinx individuals who had not received COVID-19 vaccinations and were hospitalized for COVID-19 on vaccine decisions?

**Findings:**

In this qualitative study consisting of interviews with 25 Latinx individuals who were unvaccinated and hospitalized with COVID-19, participants described the impact of hospitalization on their vaccine deliberation. After hospitalization, Latinx individuals who remained undecided and those who ultimately accepted the COVID-19 vaccine described COVID-19 vaccine concerns, and those who were vaccinated after hospitalization were motivated to engage in advocacy to encourage vaccination and suggested additional patient-centered opportunities to increase vaccine uptake.

**Meaning:**

These findings suggest opportunities to support culture- and language-concordant interventions to increase patient advocacy, address mistrust and social media misinformation, and overcome barriers to vaccine access.

## Teaser

This qualitative study investigates the perspectives on vaccination of Latinx individuals who had not received a COVID-19 vaccine and were hospitalized with the infection.

## Introduction

COVID-19 has disproportionately impacted certain racial and ethnic groups that also face a disparate burden of socioeconomic and structural health challenges.^1^ Throughout the COVID-19 pandemic, Latinx (includes Hispanic and is the preferred non–gender-based term for Latino or Latina individuals) in the United States have experienced a 1.1-fold to 7.7-fold higher risk of infections, 1.5-fold to 4.0-fold higher risk of hospitalizations, and 3.2-fold higher risk of deaths compared with non-Latinx White individuals.^2,3,4^ There are an estimated 62 million Latinx individuals in the US, representing 19% of the US population, and nearly one-third of Latinx individuals report limited English proficiency, while 23% are foreign-born and 13% are undocumented.^5,6^ Compared with non-Latinx White individuals, Latinx individuals, particularly those who are immigrants, are more likely to lack health care access and report factors associated with increased COVID-19 exposure, such as living in poverty, using public transportation, being employed in the essential workforce, and residing in high-density housing. They are also more likely to have chronic conditions associated with increased risk of developing severe COVID-19 infection compared with non-Latinx White individuals.^7,8,9^

Almost 2 years into the COVID-19 pandemic, an estimated 74% of the US population has received at least 1 dose of a COVID-19 vaccine. While disparities in uptake are narrowing, serious inequities remain.^10^ For instance, in Colorado, 40% of Latinx individuals vs 77% of non-Latinx White individuals have received at least 1 COVID-19 vaccine dose as of April 21, 2022.^11^ Previous studies^12,13,14,15,16,17,18,19^ of Latinx perspectives have reported COVID-19 vaccine deliberation concerns, including mistrust and perceived discrimination, as well as vaccine misinformation. As new COVID-19 variants emerge and disparities persist, there is a critical need for evidence to guide vaccine outreach strategies that meet community needs.

To our knowledge, no study has assessed the perspectives of Latinx individuals who were unvaccinated and subsequently hospitalized for COVID-19 pneumonia regarding the COVID-19 vaccine and how this experience is associated with vaccine deliberation. In this study, we describe Latinx perspectives on the COVID-19 vaccine after hospitalization.

## Methods

The Colorado Multiple Institutional Review Board reviewed and approved this qualitative study. Participants provided verbal informed consent to be interviewed and permission to publish deidentified quotations. We used the Consolidated Criteria for Reporting Qualitative Research (COREQ) reporting guideline to report this study.

### Study Design, Participants, and Settings

We conducted semistructured interviews. Eligible participants included English-speaking or Spanish-speaking adults aged 18 years or older who self-identified as Latino, Latina, or Latinx or Hispanic in Denver, Colorado, and who had not received COVID-19 vaccine shots prior to a 48-hour or longer hospitalization at an urban safety net hospital from February to November 2021. Using *International Statistical Classification of Diseases and Related Health Problems, Tenth Revision *(*ICD-10*) codes, we identified participants who had a principal diagnosis for COVID-19 (*ICD-10* U01.1) and a secondary diagnosis of pneumonia (*ICD-10* J12.89), acute respiratory distress syndrome (*ICD-10* J80), or acute respiratory failure (*ICD-10* J96.0). We identified participants via a data query that provided contact information for individuals who met eligibility criteria. Purposive sampling was used to capture a diverse sample in terms of sex. An interviewer (L.C., D.M., R.I.P., L.J.P., and S.A.S.) called participants by telephone 2 times within 9 months after hospital discharge. To avoid creating or exacerbating power dynamics, questions were open ended and nonleading and participants were informed that there were no right or wrong answers and reassured that their participation would not affect the health care they received.

### Data Collection

Semistructured telephone interviews were conducted by authors (L.C., D.M., R.I.P., L.J.P., and S.A.S.) in English or Spanish by telephone. Interviewers included internal medicine hospitalists (L.C., D.M., and S.A.S), an endocrinologist (R.I.P.), and an epidemiologist (L.J.P.) No interviewers had previous clinical interactions or relationships with participants. The interview guide (eTable in the Supplement) was informed by a review of the literature.^7,13,14,15,17^ Interviews were audio recorded, transcribed, and conducted until thematic data saturation was reached. Data saturation was defined as the end point in the data-collection process when no new information was discovered in thematic analysis.^20,21^

### Data Analysis

Interview transcripts were imported into Atlas.ti version 22 (Atlas.ti Scientific Software Development).^22^ Authors (L.C. and C.A.H.) performed line-by-line coding to identify preliminary concepts using inductive and deductive theme approaches. Preliminary codes were also based on research priorities and questions. Coding structures were then refined to capture emerging themes, which were grouped into subthemes using principles of grounded theory and thematic analysis. Authors assessed each analysis for interrater reliability and reached consensus with authors D.M., R.I.P., L.J.P., S.A.S., and J.F.S. Investigator triangulation was performed to ensure that themes reflected the full range and depth of the data. Member checking was not conducted.

## Results

A total of 25 Latinx adults (14 [56.0%] women, 11 [44.0%] men; mean [SD] age, 51 [15] years) participated (Table 1).^23,24^ There were 15 participants (60.0%) who preferred to conduct the interview in Spanish, and 11 individuals relied on Emergency Medicaid, which is the hospital coverage for immigrants in Denver who are undocumented. There were 10 individuals (40.0%) who were essential workers and 13 individuals (52.0%) who were unemployed. Participants were hospitalized for a mean (SD) 8 (9) days. After hospitalization, 18 individuals had received the COVID-19 vaccine, 2 individuals were in the process of scheduling their vaccines, and 4 individuals remained undecided. Participants who reported that they would accept the vaccine but had not yet received it were in the process of scheduling their appointment. Among eligible individuals contacted to participate, 6 individuals declined. The most common reasons for declining were “not interested” in the study (2 individuals) and “do not want to talk about the COVID-19 vaccine” (4 individuals). The mean [SD] duration of interviews was 25 [11] minutes. We identified 3 themes, each with subthemes (Table 2).

**Table 1.** Participant Characteristics

Characteristic	Participants, No. (%) (N = 25)
Demographic characteristic	
Age, mean (SD), y	51 (15)
Sex	
Women	14 (56)
Men	9 (36)
Preferred interview in Spanish	15 (60)
Married	9 (35)
Country of origin	
Mexico	15 (60)
Honduras	1 (4)
United States	9 (36)
Socioeconomic characteristic	
Completed high school education	10 (40)
Household income, $	
<25 000	17 (68)
25 000-49 999	7 (28)
50 000-74 999	1 (4)
Would accept COVID-19 vaccine in the future	
Yes	
Received COVID-19 vaccine	18 (72)
Scheduling COVID-19 vaccine	2 (8)
Unsure	5 (20)
More than 1 person with COVID-19 at home	11 (44)
Type of work	
Essential work^a^	
Critical trades^b^	10 (40)
Agriculture and food production^c^	6 (24)
Critical retail^d^	2 (8)
Transportation	2 (8)
Other	2 (8)
Unemployed	13 (52)
Stay at home	1 (4)
Paid hourly	9 (36)
Insurance type	
Emergency Medicaid^e^	11 (44)
Medicaid	5 (20)
Medicare	1 (4)
Commercial	8 (32)
Clinical characteristic	
Influenza vaccine received this year	12 (48)
Length of stay, mean (SD), d	8 (9)
Comorbidity	
BMI	
<30	7 (28)
30-34.9	10 (40)
≥35	8 (32)
Diabetes	17 (68)
Hypertension	11 (44)
Hospital course	
Acute kidney injury	5 (20)
Acute liver injury	16 (64)
ARDS	4 (16)

Abbreviations: ARDS, acute respiratory distress syndrome; BMI, body mass index (calculated as weight in kilograms divided by height in meters squared).

^a^ Essential work definition is from ISSA^23^ and the National Conference of State Legislators.^24^

^b^ Construction work and employment as electrician, plumber, or cleaning professional.

^c^ Restaurant work and animal and crop production.

^d^ Grocery and hardware store work and employment as mechanic.

^e^ Hospital coverage for Denver residents who are undocumented.

**Table 2.** Themes, Subthemes, and Illustrative Quotes

**Themes and subthemes**	**Quotes**
**Factor associated with vaccination after hospitalization**	
Fear of death	“My doctor goes, ‘And before you leave … I’m thinking about you getting the vaccine.’ I told him I was kind of iffy on that. So he asked me why, and he said, ‘I don’t know if you’ll survive if you get hit with one of the other variants.’ So I do think I’ll get the vaccine, I do. Because I have a 10-year-old son, and if anything were to happen to me, I don’t know. I mean, he has brothers and sisters and family, but nobody can take care of your child like you can.”
	“They transferred me from one room and they said, ‘I’m going to bring the machine to intubate him,’ and I thought, ‘That’s the end, I’m going to die here.’ I got scared … I thought, ‘I’m going to die.’ I didn’t believe in COVID. … Then, we got infected, and after that we got the vaccine. … My opinion changed for the best, because I said, ‘I’ll protect myself with the vaccine.’”
	“I could have lost my life to COVID, and it just changed my whole opinion about the vaccine. ... It’s not a joke. I could have ended up dying … and then my mom burying her youngest son and leaving behind my friends and family.”
	“Yesterday, a cousin of mine died of COVID. So my point of view has changed a little bit. I think people should [get the vaccine] because it will help them and it will prevent more serious problems. I fought against the infection. … I didn’t come out of it in the best condition, but I survived. My cousin didn’t. She died yesterday afternoon.”
Avoiding hospitalization and reinfection	“We were the first ones to get vaccinated … after we left the hospital. We got vaccinated because we believed the COVID virus did exist. And we were victims of that disease. So we got vaccinated because we don’t want more disease.”
	“I still believe the vaccine hasn’t been tested enough, but … they say that if you get the vaccine, you have better chances, and I don’t want to catch it again because it feels really ugly. … This is the first time in my life that I’ve been in the hospital. … What is the option to not go back to the hospital? It's to get the vaccine. … My point of view has changed a little bit. If the vaccine can help us in any way, we have to take advantage of it. … I don’t want to get infected again.”
	“The same day I left the hospital, I got vaccinated. I just had my appointment, and I thought I could get it that day. … I really want to be protected because this disease really scared me, to be honest.”
Convinced COVID-19 is real	“Actually, I didn’t believe in COVID before. Honestly, I never believed in it until all 3 of us were infected at home—my wife, my daughter, and myself. There were some coworkers who got sick, but I wouldn’t believe it. They said, ‘I got corona,’ and I would say, ‘I don’t believe in it.’ Until I caught it. … I was admitted to hospital … and nothing convinced me that COVID was real until I got infected.”
	“A doctor would not have convinced me … you have to see for yourself, seeing that 2 or 3 people have the vaccine and have resisted getting ill. If you see this, then it is logical that you could be convinced and you can see that it is good.”
Responded to pressure from others	“If I did tell anybody that I didn’t get the vaccine, then they’d have that look on their face of like, ‘You haven’t gotten the vaccine yet?’ And it’s just like, chill out. Like, OK, I get it, but I don’t need you to, like, guilt trip me or rub it in my face.”
	“I am getting the vaccine, yes, because now employers are no longer employing you if you don’t have the COVID vaccine. If it wasn’t for this, I wouldn’t get the vaccine, no.”
	“One of my daughters is very smart … they actually thought she was a doctor. She does her research on COVID. I pretty much felt pressured by my girls. But it was like, I still didn’t want to get vaccinated, believe it or not. … But the only reason I did get vaccinated was for them.”
**Concerns about COVID-19 vaccine**	
Experimental status and short timeline for production	“Everyone has a different opinion about the vaccine. Some people recommend it strongly, others are very fearful as I am. Since it’s experimental, it’s complicated to trust something that’s experimental, right? So there’s a lot of … fear, fear really.”
	“Why do they say the knowledge for this vaccine has existed for years, that they already knew how to create it? If they already had the knowledge to create this type of vaccine, then why haven’t they rolled out other types of vaccines that use the same knowledge for AIDS or pediatric cancer? Why don’t they use that same knowledge? Instead, they say, quickly, here’s the vaccine and you must all get it.”
Contents of vaccine unknown or concerning	“I was also concerned about the vaccine because I heard it had ‘graphene,’ and I didn’t understand. Why does it have those substances? Why were they insisting on people getting vaccinated if it has graphene?”
	“I don’t know why some people say they are bad, that they die, and others say it is good. In order to receive the vaccine, I need to know more about the content of the vaccines, good information, and that’s it, and about each one of the vaccines.”
Vaccine considered ineffective	“I don’t believe in that [vaccines]. I don’t believe in that. … I don’t think the vaccine works. I have a very close friend, she had COVID 3 times, and the third time she was practically paralyzed, and she had 3 doses of the vaccine. How can I have faith in the vaccine? It’s incredible to see those situations. It’s really hard, with or without a vaccine. There’s no guarantee. That’s why it doesn’t make sense to me, doctor. I’ll put my body at risk, doctor, but there’s no guarantee that I won’t get COVID. So why would I put myself at risk by introducing something that my body will react to? That doesn’t make any sense.”
	“I wasn’t a fan of the vaccine. I asked them at [the] clinic, and they said they had a high percentage of vaccinated people who were admitted with COVID. … I also heard that the vaccine was still an experiment, and so I said, ‘I will not get vaccinated.’ So the question is, why do people keep getting sick with COVID? Why do people who are vaccinated have to stay home and wear a mask if they’re vaccinated?”
Worrisome immediate and long-term adverse effects	“And it first it was just like the first couple of people receiving the shot were maybe having some adverse reactions or maybe passing out for a few minutes and that kind of thing. So that also was just a little bit of cause for concern because I haven’t had a primary care doctor in a while. … In the back of my mind, it’s also just the whole thing of like, again, trying not to be like any other person who is like super antivax, but it’s like, I also don’t want to have any like crazy adverse reactions like years later from getting the shots.”
	“I’ve heard that it causes health issues or blood clots and stuff like that because the vaccine hasn’t been developed for—it was just developed, like, recently. So no one really knows the long-term effects of the actual vaccine. That’s how I look at it. … I think it was just, like, blood clots and stuff like that, such as having heart issues, I guess, as well.”
Mixed and conflicting information	“I would just see news articles from, like, different companies and, like, saying COVID’s bad or the vaccine’s good, and then the vaccine is bad. Or, like, social media groups and such, talking about how the vaccine is not really safe and how did we get, like, a vaccine right away when this is, like, a new COVID, a new disease or sickness that’s going around. Like, how did we just get a vaccine right away?”
	“Look, I really don’t trust all of them. Not all news programs are trustworthy. It’s complicated. That’s why I mentioned there are contradictions because one channel has some information and others have different information. That’s why it’s confusing.”
Government aimed to control or mark population through vaccination	“I have conspiracy theories, you know, regarding, like, government stuff, like medications and vaccines. So I always thought the government was trying to control the population. Like, why would you get a vaccine if it’s not been out for so long? So I was always thinking, and I was like, ‘Why is the government trying to push the vaccine very hard?’ … I was thinking, like, well, ‘I don’t know if I can trust the government because the government, I guess, they hide stuff from us. So they’re pushing the vaccine out a lot more.’ So that’s where my—I start thinking about stuff in a negative way because the government’s trying to do something to the people or to us, injecting us with a whole bunch of different stuff. There’s multiple companies that make the vaccine, which is kind of weird.”
	“And about the vaccine, I think it is not good yet. I think it is something that they put on you to locate you. It’s like they are chosen in case that later on there is like a world war or something, because I think that vaccine brings something that identifies them.”
	“I do think that the government is behind all of this. This virus was planned, this virus that keeps going on and on. I can’t say for certain that it was the government, I don’t know, but this is something abnormal. This is very strange. Now that there’s going to be a fourth and fifth shot and so many vaccines … I do doubt the vaccine. … I don’t know where it comes from, what the government is doing, what purpose they have to vaccinate all the people, making it mandatory, pay for them to get vaccinated.”
**Opportunities to improve vaccine uptake**	
Sharing personal experiences on social media	“Through social media, sharing our experience where a lot of people are connected … I think WhatsApp, because many people, like me, use WhatsApp a lot to communicate with family. But also Facebook I think is a very important place for communication.”
	“Before I got sick, I just heard that the virus was around. … I mean, I knew it was real, but well, I didn’t think it was so real. A lot of people maybe think it’s all a lie. … My mom shared stuff when I got sick via Facebook, like my status and all that, my health. … She did this to share awareness that COVID is real. Because of this, my sisters in Mexico, they all got vaccinated … because, before I got sick, they weren’t fully convinced.”
Testimonials about minimal vaccine adverse effects	“Like, we could give testimonials, I think, from people who have already been vaccinated and everything has gone well for them and they haven’t had, maybe, what they think is some concern or a bad side effect.”
	“I guess you don’t have to guilt trip people, but just make them more aware of even how many other friends of mine that have gotten it and they were fine afterwards.”
Connecting with friends and family about the hospitalization experience	“Yes, I told them, take care of yourselves, get tested, because look at me, I didn’t believe in it and see where I am now. … They called me and said, ‘How are you?’ Then, I would tell them, ‘Take care of yourselves, because I didn’t believe in it, and look where I am.’ Some of my friends went to get the vaccine. My wife and daughter were also convinced about the COVID vaccine that way after I was hospitalized.”
	“I told the ones that weren’t vaccinated, and they saw me and they got their shots. I’m like living proof … and that’s what I tell my coworkers: ‘I don’t want you to go through what I’m going through. I don’t want you to go to the hospital because it’s awful. … This disease is real, not everyone gets it the same way but it’s better to be protected.’ I told them, ‘I’m in the hospital, and thank God I’m recovering, but many people don’t make it.’”
	“I’ve already shared my experience with COVID with my brother, with my friends, with my coworkers. My brother, he didn’t believe this and he didn’t get vaccinated, but when I got sick and ended up in the hospital, he saw that and got his vaccine. It’s just that when this happens to a relative, well, then you start to feel bad, this is awful. I always tell my friends what happened to me, the amount of time I was hospitalized, with oxygen, and, well, the experience I had.”
	“I tell my cousins the truth, what happened to me in the hospital and why it happened to me, and I tell them that I needed to get vaccinated so that it wouldn’t happen to me again. I haven’t asked them if they have already been vaccinated or not, but when I talk to them, I try to more or less convince them, to make them think that it is necessary. This happened to me, and something serious could happen to them.”
Making the vaccine more accessible	“Sometimes, you say, ‘Yes, I’m going to do it.’ Maybe I should have been more careful about finding the time. But also, maybe it would have been helpful to have more information about closer locations. Maybe not having to make an appointment for the vaccine and instead just going and it would be faster.”
	“If someone came to vaccinate everyone at work, it would help. … Sometimes, we can’t leave work. I mean, it’s OK to miss a day or 2 days, but we need to let our employer know in advance. … That’s the way it is. If I had my own job, I would say, ‘I’m not going to work today because I have to get vaccinated in the morning.’”
	“I did call to make an appointment, and they talked to me in English. I don’t know if they didn’t have a Spanish speaker at the time, but I didn’t know. … I didn’t understand well enough.”
Connecting with trusted sources of information	“Just talking to the doctor, I guess, would have helped me or benefited me more. Yeah, understanding side effects and how this is going to affect me down the road when I’m older. I wish that I would have known. I think if I had a little bit more knowledge about the vaccine that I would have made the decision to get vaccinated sooner.”
	“I think that nowadays everything is commercial, for sales purposes, everything is fake or manipulation. It’s like, it’s difficult to find real information. … I’m more open now, though, to get information, more than anything, more sources of information that go in depth. But it’s going to be very difficult for me to find information that tells the truth because all of them say, ‘Don’t worry, it’s going to be fine,’ and then people that are vaccinated die and unvaccinated people die, young people die, old people continue to die. There are too many questions science hasn’t answered.”
	“Knowing more about the COVID-19 disease would have helped. It would help more, and everyone would be a little more aware of getting vaccinated, so that they don’t get as sick as I did.”

### Factors Associated With Vaccination After Hospitalization

#### Fear of Death

For some patients, “surviving COVID” was a close brush with death and the fear of death outweighed concerns about the vaccine. One participant said, “They said, ‘I’m going to bring the machine to intubate him,’ and I thought, ‘That’s the end, I’m going to die,’ and my opinion changed for the best because I said, ‘I’ll protect myself with the vaccine.’” Participants were worried about leaving family members behind. One participant stated, “I have a 10-year-old son, and if anything were to happy to me, I don’t know.” Another stated, “I could have ended up dying and then my mom burying her youngest son and leaving behind my friends and family.”

#### Avoiding Hospitalization and Reinfection

After hospitalization, participants described being the first ones among their friends or family to be vaccinated. Participants described how their motivation was to “not go back to the hospital … my conscience said, ‘Do it and get it [the vaccine] over with.’”

#### Convinced COVID-19 Is Real

Participants and their family or friends who were unsure if the COVID virus was real were convinced by the participant’s hospital experience that COVID was real. One participant said, “I don’t think you can convince people until they are infected with COVID, like a relative, someone close to them, because, like I said, Hispanics don’t believe … you don’t trust anyone, and you don’t believe things until they happen to you.” Some participants were convinced by seeing other people who were sick in the hospital. Another participant said, “I went to the hospital, and it was full, people were intubated, and yes, it’s all true, it’s true. I saw all of that. I mean, being there changed my mind so much, sincerely.”

#### Responded to Pressure From Others

Some individuals who were reluctant to receive the COVID-19 vaccine after their hospitalization described feeling “forced” to be vaccinated because their employers required it or because they felt “pressured” to receive it by their family. One participant said, “Oh, I still don’t trust the vaccine. I was pressured to get the vaccine by my daughters, who said, ‘Oh, you’re selfish if you don’t do it.”

### Concerns About the COVID-19 Vaccine

#### Experimental Status and Short Timeline for Production

Participants who remained undecided about the COVID-19 vaccine after hospitalization and those who ultimately accepted the vaccine were worried by how quickly the vaccine became available and wondered if it was “just an experimental vaccine.” These concerns were encouraged and amplified by their friends and trusted sources in the community: “People from the church, priests, too, have said that to create a good vaccine that won’t harm you, years have to pass, and they say that this vaccine was created too quickly.”

#### Contents of Vaccine Unknown or Concerning

Concern for “what exactly is in the vaccine in terms of ingredients” or fears that ingredients may be harmful were common. One participant said, “I know that within the first year, there was speculation about how no one could ever actually tell you what the ingredients in the vaccine were.”

#### Vaccine Considered Ineffective

Participants recounted stories of family and friends who had been infected with COVID-19 despite being vaccinated and expressed concerns that the “vaccine does not work.” One participant said that despite being hospitalized, he would not be vaccinated in the future unless convinced that the vaccine was effective in preventing infection: “Even my son had the COVID vaccine, and he got COVID so I didn’t get vaccinated. … I may get vaccinated in the future, but the only thing that would convince me is if it has a result, meaning that people don’t get infected with COVID.”

#### Worrisome Immediate and Long-term Adverse Effects

Social media frequently informed beliefs about potential vaccine adverse effects: “I’ve seen plenty of videos of people getting so ill. … I was terrified to get the vaccine … videos of people who can’t walk.” Participants worried about adverse effects that they had heard about through word of mouth from friends and family: “They [friends and coworkers] said they were going to become infertile, that they wouldn’t be able to have kids.”

#### Mixed and Conflicting Information

Participants described inconsistent messaging about vaccines and wondered which media sources were reliable: “We had misleading information … because some news outlets say one thing and others say other things.” The confusion came when hearing differing messages from TV, social media, and clinicians: “As I said, some doctors say it is not advisable, and others say it is … then you get confused. If the doctors say one thing, and others say something else, imagine for us, the people who don’t even know.”

#### Government Aimed to Control or Mark Population Through Vaccination

Participants had heard through word of mouth and social media that the vaccine was created by the government to control people: “I heard that they want to dominate us and control us … that they were going to insert a chip in the vaccine.” Another participant said, “It was all over the internet that COVID was a way to affect the economy and to put a chip in our arm to control us.”

### Opportunities to Improve Vaccine Uptake

#### Sharing Personal Experience Through Social Media

Participants who had received most of their COVID-19–related information on social media described sharing some of their personal hospital experiences through social media. They said this was done to convince family and friends.

#### Testimonials About Minimal Vaccine Adverse Effects

Realizing that many of their friends and family were concerned about COVID-19 vaccine adverse effects, participants who had been vaccinated reported sharing personal experiences of minimal adverse effects to allay fears. One participant said, “If I see others who don’t want to get the vaccine, I say that I already got it and nothing happened to me.”

#### Connecting With Friends and Family About the Hospitalization Experience

Participants who were vaccinated after hospitalization intentionally reached out to friends and family who were unvaccinated to share their hospital experience and convince them to be vaccinated: “Two of my friends listened, and they went ahead and got vaccinated. I told them I was hospitalized and that I needed oxygen, so they said, “Then, it’s a b--ch. Let’s get vaccinated.” Participants described how it was particularly important to share their experience because they had relied on COVID-19 information through word of mouth and their neighbors continued to rely on COVID-19 information through word of mouth: “All the people talked about how getting the COVID-19 vaccine led to death … there are neighbors here that aren’t vaccinated yet because of that … it was spread through word of mouth.” Some participants described a mixed response from friends and families, with some immediately getting vaccinated while others remained hesitant about the vaccine.

#### Making the Vaccine More Accessible

Participants admitted that they considered getting the vaccine but just “didn’t really make the time,” as 1 participant said, because there were always “so many things to do at home … and then it gets overlooked.” Some participants had competing priorities, such as childcare or caregiving for ill family members, that made it difficult for them to attend an appointment or wait in line for the vaccine. Those who had not been vaccinated owing to competing priorities or social challenges described how making the vaccine more accessible in their community setting or place of work would have been helpful. Increasing the availability of language-concordant staff was also described as helpful by participants who reported limited English proficiency and were not vaccinated because no Spanish speaker was available to help them with their appointment.

#### Connecting With Trusted Sources of Information

After hospitalization, participants felt they needed more information for vaccine deliberation. They also felt it would be helpful to have conversations with trusted sources, such as faith-based leaders, or access websites that provided accurate and consistent information. One participant said, “I need to do more research on the vaccine … maybe talk to more people about the vaccine … my pastor.”

## Discussion

In this qualitative study, we sought to understand the perspectives of Latinx individuals who were unvaccinated and subsequently hospitalized for COVID-19 and how their hospitalization was associated with their vaccine deliberation. After hospitalization, most participants were vaccinated because they were motivated by fear of death and the desire to avoid reinfection and rehospitalization and because they were convinced that COVID-19 was real. Participants who remained unsure about receiving the COVID-19 vaccine described concerns about its experimental status, unknown contents, perceived ineffectiveness, and adverse effects; receiving mixed information; and the involvement of government. Participants who received the vaccine after hospitalization had engaged in personal advocacy to encourage vaccination among friends and families and had thoughts on how to improve vaccine uptake.

Latinx adults in our study described personal advocacy efforts by sharing about their experience of hospitalization through social media and word of mouth with other individuals who were unvaccinated. These outcomes suggest that hospitalization may have been a defining experience that motivated individuals to engage in advocacy. Our findings suggest that hospital discharge and the postdischarge period may be critical times to provide interventions to increase vaccine uptake. A public health strategy could include providing social media support for Latinx individuals who are being discharged from the hospital and who are interested in sharing their experience. This could include providing assistance with taking a photograph or recording a video, suggestions for how to describe the COVID-19 virus and vaccine, and trusted links to tag with COVID-19 information and identifying a clinician who could be tagged and answer questions on their social media page. Another strategy may be to provide individuals being discharged with a phone number they could call for future guidance on how to respond to questions about the COVID-19 vaccine. Latinx individuals in our study also described how their motivation to increase vaccine uptake among peers activated them to share the minimal adverse effects they experienced with the COVID-19 vaccine. A recent COVID-19 vaccination intervention in Latinx individuals^25^ included a component of activation that emphasized patients’ willingness and ability to take independent actions to manage their health and care and encouraged Latinx individuals who were vaccinated to act as ambassadors to increase vaccine uptake. Clinicians who are members of racial and ethnic minority groups have also increased their visibility on social media to share vaccine information.^26,27,28,29^ A potential public health strategy to increase the social media presence of racial and ethnic minority clinicians may be to provide protected time and social media tools to amplify expert messaging around the benefits of COVID-19 vaccination.^30^ Lastly, continued advocacy efforts that urge the government to ensure that COVID-19 misinformation be removed from social media are critical.^31,32,33,34,35^

Latinx individuals in our study described the need to connect with trusted sources of information about the COVID-19 vaccine. Participants with COVID-19 vaccine concerns reported that the government aimed to control or mark the population through vaccination. The association of government mistrust with health behaviors has been described among Latinx individuals who had COVID-19 and waited longer to seek medical care than non-Latinx White individuals.^7,14^ The public charge rule change (a law that penalized use of public benefits by immigrants who sought permanent legal residency) went into effect in February 2020, just weeks after the first confirmed COVID-19 infection in the US.^36^ Compounding the anti-immigrant policies and rhetoric were reports of continued threats by Immigration and Customs Enforcement despite public announcements that it would cease civil immigration enforcement during the pandemic.^37^ To mitigate mistrust and increase vaccine uptake, an important strategy may be to employ trusted community health workers who encourage unrushed, empathic, and bidirectional conversations about the COVID-19 vaccine.^38,39,40,41^ With new variants emerging, more resources and funds are needed to support community health worker interventions throughout the country. Another promising strategy may be to partner with faith-based leaders who are trusted information sources.^42,43^ Interventions engaging faith-based communities have been associated with improved vaccine uptake.^44,45,46^

In our study, some participants wanted the vaccine but because of a lack of available language interpreters or the presence of competing social challenges, were not vaccinated. These findings are consistent with a survey^47^ that assessed a diverse cohort of individuals who were unvaccinated and found that Latinx individuals were 2-fold as likely to say that they would like to be vaccinated as soon as possible compared with non-Latinx White individuals. Latinx individuals are more likely to face social challenges, and ensuring vaccine opportunities that are language and culturally concordant, as well as convenient, may continue to narrow vaccine uptake disparities.^25,48^ Some social and logistics barriers that Latinx patients reported have also been reported by other racial and ethnic groups.^12,15^

### Limitations

Our study has several limitations. Authors who interviewed participants noted that, compared with participants in previous qualitative studies they had conducted, participants in this study were reticent to share their perspectives. To overcome mistrust, interviewers spent the first few minutes of each interview in a conversation unrelated to COVID-19. Despite efforts to build trust, social desirability bias and mistrust may have led some participants to censor negative views about the COVID-19 vaccine. Additionally, we recruited participants from a single hospital, where the Latinx population is primarily from Mexico. Transferability of the findings to other health systems or Latinx cultures is unknown.

## Conclusions

In this qualitative study, English- and Spanish-speaking Latinx individuals who were unvaccinated described the impact of hospitalization for COVID-19 on their vaccine deliberation and their personal advocacy to encourage vaccination in their communities. Our findings suggest the need to fund and support culture- and language-concordant interventions that may increase patient advocacy, address mistrust and social media misinformation, and overcome barriers to access by bringing the COVID-19 vaccine to the community.

## Notes

Supplement. **eTable.** Interview Guide
