# Error in Results

**DOI:** 10.1001/jamanetworkopen.2022.23980

**Published:** 2022-07-12

In the Original Investigation titled “Perspectives of Latinx Individuals Who Were Unvaccinated and Hospitalized for COVID-19: A Qualitative Study,”^1^ published June 17, 2022, there was an error in the Results. The number of individuals who remained undecided after hospitalization should have read 5. This article has been corrected.^1^
