# Supplementary material for: Perspectives of Latinx Individuals Who Were Unvaccinated And Hospitalized for COVID-19: A Qualitative Study
Source: JAMA Netw Open. 2022 Jun 17;5(6):e2218362. doi: 10.1001/jamanetworkopen.2022.18362 (PMC9206184; doi:10.1001/jamanetworkopen.2022.18362)
Supplement: Supplement. — eTable. Interview Guide [file jamanetwopen-e2218362-s001.pdf]

Supplemental Online Content

Cervantes L, Hazel CA, Mancini D, et al. Perspectives of Latinx individuals who were unvaccinated and hospitalized for COVID-19: a qualitative study. *JAMA Netw Open*. 2022;5(6):e2218362. doi:10.1001/jamanetworkopen.2022.18362

eTable. Interview Guide

This supplemental material has been provided by the authors to give readers additional information about their work.

© 2022 Cervantes L et al. *JAMA Network Open.*

eTable. Interview Guide

1. Prior to your admission to the hospital, what were your thoughts about the COVID-19 virus and the the COVID-19 vaccine?<br><br>2. What were you hearing about the COVID-19 vaccine and the COVID-19 virus from friends and family?<br><br>3. What questions did you have about the COVID-19 vaccine?<br><br>4. Is there information that might have helped you make a decision about the COVID-19 vaccine?<br><br>5. How has being in the hospital with COVID-19 changed or not changed how you feel about the COVID-19 vaccine?<br><br>6. Have you shared your experience of being hospitalized with COVID-19 or being vaccinated with COVID-19?

© 2022 Cervantes L et al. *JAMA Network Open.*
